# A Review on the Incidence and Related Risk Factors of Retinopathy of Prematurity Across Various Countries

**DOI:** 10.7759/cureus.32007

**Published:** 2022-11-29

**Authors:** Arun Nair, Ruaa El Ballushi, Burjor Z Anklesaria, Maryam Kamali, Mashal Talat, Tabitha Watts

**Affiliations:** 1 Pediatrics, Saint Peter’s University Hospital, New Brunswick, USA; 2 School of Medicine, Royal College of Surgeons in Ireland - Medical University of Bahrain, Muharraq, BHR; 3 Family Medicine, Dubai Health Authority, Dubai, ARE; 4 Medicine, Manchester University National Health Service Foundation Trust, Manchester, GBR; 5 Pediatric Emergency Medicine, John H. Stroger, Jr. Hospital of Cook County, Chicago, USA

**Keywords:** pediatrics and neonatology, childhood blindness, prematurity, oxygen therapy, retinopathy of prematurity

## Abstract

Retinopathy of prematurity (ROP) is an ophthalmologic condition that is one of the leading causes of preventable childhood blindness. Due to the premature nature of blood vessels in preterm infants, retinal vessels are prone to damage. The incidence of ROP ranges with great variation across countries, and this study aims to identify the incidence and its related risk factors.

A compilation of studies investigating the incidence of ROP was acquired through the PubMed and Google Scholar databases with a full free text in English filter set. All members of the study were involved in designating studies based on continent and arranging them into a table format. Following this, reasons for the variation in the incidence of ROP were investigated by individually assessing each study.

The variation of an increased incidence of ROP seen in low-income countries (LICs) was most likely due to the availability of resources. As a preterm birth can give rise to further complications such as the development of sepsis, it is important to manage preterm birth with the utmost caution. Evidence has suggested that the two key variables in reducing the morbidity and mortality of ROP are the implementation of a screening and treatment protocol with controlled use of oxygen and the availability of resources in hospitals to adequately identify and manage ROP as early as possible.

Through a comprehensive overview of the incidence of ROP, it is vital to review screening and treatment protocols in each hospital for ROP, which would aid in preventing its occurrence and initiating early treatment.

## Introduction and background

Retinopathy of prematurity (ROP) is identified by the World Health Organization (WHO) as a major cause of preventable childhood blindness worldwide [1]. It is a disorder of retinal blood vessels in which they are developed poorly in preterm infants with low birth weight [2]. Normal retinal vasculature development begins at around 16 weeks of gestational age around the optic nerve and continues through pregnancy. The optic structures and vasculature are normally fully developed in term infants, which is why retinopathy of prematurity is rare in this case. It mainly occurs in premature infants with incomplete development of retinal vasculature [2,3]. It is classified depending on the affected zone and the stage of the disease according to the International Classification of Retinopathy of Prematurity (ICROP) to assess its level of severity [3].

Approximately 14,000 preterm infants in the United States alone develop retinopathy of prematurity, and between 1,100 and 1,500 cases of them progress into moderate to severe stages of the disease that requires medical attention and treatment [2]. Long-term consequences of retinopathy of prematurity include myopia that can be severe and associated with strabismus, as well as macular scarring, cataracts, glaucoma, and/or retinal detachment. Despite the intensive care and medical treatment provided to them, around 400-600 of the affected infants become completely blind from this condition in the United States alone [2]. Individuals with a prior history of retinopathy of prematurity require life-long ophthalmology surveillance and screening for complications [2].

There are multiple risk factors affecting the development and progression of retinopathy of prematurity, including low birth weight, preterm gestational age, appearance, pulse, grimace, activity, and respiration (APGAR) score, and especially the use of supplemental oxygen, which causes downregulation of the vascular endothelial growth factor (VEGF) due to oxidative stress and death of endothelial cells, intraventricular hemorrhage, sepsis, blood transfusions, and most importantly, lack of timely screening [3]. Retinopathy of prematurity can actually be a strong measure of the quality of healthcare services that infants receive in the healthcare institute [4].

Management strategies vary depending on the ICROP classification system. Current treatment guidelines include laser peripheral retinal ablation for the early stages of the disease. Anti-VEGF inhibitors such as bevacizumab have also recently emerged with promising results, especially in the early stages of the disease. Further information and research are needed on this novel therapy as there are insufficient data on its long-term effects on infants [3]. It is crucial to recognize and treat retinopathy of prematurity in its early stages by achieving the best possible perinatal and postnatal care to reduce morbidity and prevent complications [2,3]. Late presentation for screening is the most common cause of ROP-related blindness, along with false screening methods [5].

This study is conducted to estimate the incidence of retinopathy of prematurity and evaluate the pattern, prevalence, risk factors, and complications to achieve a better understanding of the disease and its burden to help healthcare professionals design efficient preventive measures and authorize setting basic minimum requirements for screening preterm and low-birth-weight infants for this condition to decrease its associated morbidity to accomplish a high and healthy quality of life for future babies.

## Review

Worldwide incidence and prevalence

The worldwide prevalence of ROP can be difficult to estimate due to not only a large number of patients but also a lack of standardized ROP screening protocols worldwide, which would enable a global registry to collect more accurate data. As seen in Table 1, only a few countries such as Kenya, Nigeria, India, the United States, and Romania, for example, have incorporated a screening protocol for ROP. ROP was most prevalent in preterm or low-birth-weight infants in most, if not all, studies, and it is imperative to note the varying risk factors that can be controlled to prevent the progression of ROP into severe visual impairment or blindness.

**Table 1:** General information on the prevalence of ROP from various countries across the world ROP: retinopathy of prematurity, CRYO-ROP: Cryotherapy for Retinopathy of Prematurity

Country	Notes
United States [2]	A total of 14,000 preterm infants are diagnosed with ROP annually with 7.8%-10.7% of patients developing a disease severe enough that requires treatment. The CRYO-ROP Group Study reported that 66% of infants with a birth weight of less than 1,250 g and 82% of infants with a birth weight of less than 1,000 g had ROP, and 400-600 infants per annum become blind due to ROP.
Iran [6,7]	The overall incidence was reported to be 23.5% according to a study by Azami et al. in 2018 with the highest prevalence reported in ROP stage 2, followed by stage 1. The prevalence was shown to decrease from 77.9% in the 24-25 weeks of gestation group to 1.1% in the 30-31 weeks of gestation group.
	In a study conducted by Zarei et al. in 2019, the prevalence was calculated to be 27.28%, of which 23.52% had bilateral disease. Out of 543 patients in this study, 223 were born extremely premature, with an average gestational age of 27.24 weeks and an average birth weight of 1,086.5 g; of these patients, only 25% did not develop ROP.
Kenya [8,9]	The prevalence was reported to be 41.7%. The majority were identified as stage 1 or stage 2 ROP. The prevalence of vision-threatening ROP was deemed to be 20.9%.
	The prevalence was reported to be 16.7%. Screening guidelines were implemented in 2018.
Nigeria [9,10]	The prevalence was reported to be 47.2%.
	The study conducted in 2015, after Adio et al. in 2014, reported that the prevalence of ROP was 15%, and half of these cases required treatment.
Sweden [11,12]	The prevalence of ROP was 18% in infants born with a birth weight of less than 1,500 g and 24% in those with a birth weight of less than 1,000 g. It was reported that the incidence can be as high as 61% in an infant born with a gestational age under 27 weeks.
	Of patients born under the gestational age of 27 weeks, 73% were reported to have ROP, with 35% of these patients being classified as having severe ROP.
Norway [12]	ROP was reported to be prevalent in 33% of patients born under the gestational age of 28 weeks.
Belgium [12]	Severe ROP was reported in 26% of patients born under the gestational age of 27 weeks.
Australia and New Zealand [12]	Of infants born under 29 weeks of gestation, 10% were diagnosed with severe ROP.
Austria [12]	Severe ROP was seen in 16% of patients born under the gestational age of 27 weeks.
Finland [12]	Severe ROP was seen in 5%-10% of patients born weighing less than 1,000 g.
India [5,13,14]	Variations in ROP prevalence were seen between urban and rural areas. In Madhya Pradesh, the prevalence was noted to be 30.1%, of which 15.8% were classified as mild ROP and 14.2% were classified as severe ROP. Aggressive posterior ROP was found in 27.7% of cases, and 83.3% of cases were identified in infants born under the age of 32 weeks of gestation. Also, 2.2% of cases progressed to blindness and/or severe visual impairment. The incidence of ROP across India range between 20% and 51.9%. The rate of severe ROP ranges between 4.7% and 13.2%.
	The prevalence was reported to be 32.6% in this study, which was conducted in Tamil Nadu. The majority of patients had bilateral disease. The highest incidence was recorded in infants born weighing less than 1,000 g. Babies born under 28 weeks of gestation had the highest incidence of ROP at 62.8%. Treatment was indicated in 13.2% of patients.
	The incidence of ROP ranged from 38% to 47% in different regions. Of the patients, 80% had spontaneous regression, and 1.8% had severe ROP.
Philippines [14]	The Philippines was classified as a high-risk nation for ROP blindness. The prevalence ranged between 14% and 33%.
Venezuela [14]	Of the patients in this study, 15.5% (69 out of 445) were diagnosed with ROP, of which 35 patients required laser photocoagulation.
Romania [14]	An ROP screening program was introduced in 2002. The ROP incidence was estimated to be between 40% and 50%.
Thailand [14]	The prevalence was last estimated between 2006 and 2010. The estimated prevalence was 40.7% in preterm infants.
Turkey [15]	The prevalence was noted to be 27.7%, and 6.7% of cases were reported to be severe. The majority of cases were reported to be prevalent in private hospitals.
Egypt [9]	No screening criteria or guidelines for ROP exist as of 2019. The incidence ranged from 19.2% to 69.4%.
Rwanda [9]	The prevalence was last estimated between 2015 and 2016 and was estimated to be 14.9%; however, the study was deemed to have a decreased estimate of the prevalence due to a high dropout rate and a wider inclusion criterion.
South Africa [9]	ROP screening protocol was implemented in 2013. The prevalence was reported to be 19.2%. Between 2011 and 2015, the prevalence of treatable ROP reduced from 8.75% to 2.36%.
Sudan [9]	The prevalence was last estimated between 2012 and 2013 and was reported to be 37%.
Indonesia [16]	The prevalence of ROP in infants who weighed less than 1,500 g at birth and were born under the gestational age of 32 weeks was reported to vary between 11.9% and 30.5%. Severe stages of ROP were found to be present in infants born up to 35 weeks of gestation and weighing up to 2,000 g at birth. In Indonesia, ROP was seen more frequently in infants with higher birth weight and gestational age.
Brazil [17]	The incidence of ROP was reported to be 33.9% with the majority (26.6%) having been diagnosed with stage 1 ROP. Out of 520 neonates who were born under 32 weeks of gestation or with a birth weight under 1,500 g, 37.6% developed ROP.
Ghana [18]	The incidence of ROP was reported to be 13.7%. All except one case had a birth weight of less than 1,500 g, and 38.9% of ROP cases had a gestational age of less than 32 weeks. Inconsistent screening of patients was noted.
Saudi Arabia [19]	Of the patients in this study, 33.3% were diagnosed with ROP. The gestational age was 32 weeks or less in 91.7% of the cases, and 72.2% of patients had a birth weight reported between 1,000 g and 1,500 g.

Risk factors

Retinopathy of prematurity has several risk factors that determine its course and presentation. These risk factors can be interlinked with the pathogenesis of the development of the different stages of the condition. Two key identifiable risk factors are infants being born at an early gestational age of less than 30-32 weeks and a low birth weight of less than 1-1.5 kg [20].

The amount of oxygen received by the child and the duration of ventilation are examples of interlinked risk factors. This could be due to the prolonged use of nasal continuous positive airway pressure (CPAP). Exposure to oxygen for longer than seven days with a saturation of greater than 50% leads to the formation of oxygen free radicals, which in turn leads to the development of ROP [6]. There has been a high rate of ROP in India due to prolonged oxygen exposure and infection development in infants. This is coupled with a lack of awareness about the condition itself and a lack of screening protocols [4].

Pulmonary conditions such as neonatal respiratory distress syndrome (NRDS), if left untreated, can lead to advanced stages of ROP. NRDS, along with early gestational age, is associated with the development of aggressive posterior ROP [5]. In respiratory distress syndrome (RDS), the infant may require oxygen therapy and mechanical ventilation, both of which are risk factors. The presence of other pulmonary diseases such as pneumonia and bronchopulmonary dysplasia leads to the development of ROP by causing intermittent hypoxia in the infant [21]. In terms of hematologic factors, repeated blood transfusions, erythropoietin (EPO) levels, and anemia are interlinked in terms of pathogenicity. Blood transfusions and EPO are usually used to treat or prevent anemia in a child, but this results in a high iron load and the formation of oxygen radicals, which causes retinal damage. Low levels of EPO lead to the weakening of the blood vessels because it is a pro-angiogenic factor.

Late-onset sepsis, including perinatal infection and inflammation, affects retinal angiogenesis via the formation of cytokines and endotoxins, which leads to retinal ischemia due to the impairment of tissue perfusion [15]. The increased oxygen demand during sepsis along with the toxic effects of high oxygen production during transfusion increases the risk of retinopathy of prematurity.

The two other factors associated with retinopathy of prematurity are poor postnatal weight gain and slower growth velocity. Slow weight gain causes a lower increase of an anabolic hormone used in the development of blood vessels called insulin-like growth factor (IGF-1) [21]. Therefore, a decrease in the level of the hormone halts retinal blood vessel growth in the newborn. Human breast milk contains immunogenic factors such as IGF-1 and epidermal growth factor (EGF-1). A shorter duration of breast milk feeding along with premature birth causes deprivation of the maternal immunogenic factors, which effectuates the hypoxic and hyperoxic phase of retinopathy of prematurity [22]. Twins or multiple births are associated with a higher risk of the baby being born preterm and of small birth weight. Discordant twin pairs with lower birth weights are more prone to develop any stage of ROP. The advanced stage of ROP is seen more in higher-birth-weight twin pairs [23].

Chorioamnionitis is a bacterial infection that occurs before or during labor, which affects the chorion and the amniotic fluid. It also induces a fetal inflammatory response causing the release of the inflammatory cytokines that affect retinal angiogenesis. If this occurs in the presence of funisitis (inflammation of the connective tissue of the umbilical cord), then this increases the chance of developing ROP. Infants exposed to chorioamnionitis are found to be born preterm, have a lower birth weight, be exposed to antenatal corticosteroids, have development of early- or late-onset sepsis, and be subjected to higher rates of partial rupture of membranes (PROM). All of these are contributing factors to the development of ROP [24].

Pre- and postnatal risk factors are very significant in the development and progression of retinopathy of prematurity. Some of these have already been mentioned and discussed above. Postnatal factors include prolonged oxygen exposure, sepsis, necrotizing enterocolitis, and intraventricular hemorrhage [25]. Necrotizing enterocolitis has the same pathophysiology as chorioamnionitis, which involves the inhibition of retinal angiogenesis. A low APGAR score at five minutes of birth has been found to be a risk factor for the development of ROP to the stages that require treatment.

Prenatal risk factors include prolonged rupture of membranes, preterm premature ruptures of membranes, hypertensive disorders of pregnancy, and chorioamnionitis. Hypertensive disorders of pregnancy are known to cause an increase in antiangiogenic factors, which antagonizes the action of vascular endothelial growth factor 1 (VEGF-1) responsible for the growth and permeability of the blood vessels. The use of beta-blockers and antihistamines in the later stages of pregnancy are also known risk factors.

The care given in higher-level hospitals or study centers of large clinical trials has shown lower rates of retinopathy of prematurity. This is due to the availability of more experienced staff able to manage premature infants [21]. Race also plays a role in the development of ROP as black infants have a lower incidence of ROP as compared to white infants [8]. The severe form of retinopathy of prematurity is found to be present in larger and more mature babies conceived through artificial reproductive technology, mainly through in vitro fertilization (IVF) [26].

Global relevance of retinopathy of prematurity

Retinopathy of prematurity is a condition in which it has been shown that with adequate screening and timely management, the progression of the disease and mortality can be drastically reduced. One of the most commonly identified reasons for increased mortality for this condition was a lack of screening protocols and utilities in certain countries. In a study conducted by Onyango et al. in 2018 [8], the prevalence and risk factors of ROP were studied in Kenya, which supported the ideology that middle- and high-income countries with advanced neonatal care have a decreased mortality with regard to ROP. This paper mentioned that previous studies in Kenya, such as that by Wanjala et al. in 2007, had shown a mortality rate of 24.5%, whereas the study by Onyango et al. showed a mortality of 4.6%. Another important point to note from this study is that there may be a low prevalence of ROP found in Sub-Saharan countries, which may be masked by the fact that there is high neonatal mortality. Factors contributing to this increased rate of mortality include a lack of advanced neonatal care for low-birth-weight and premature neonates.

There has been a significant progress in the reduction of neonatal mortality rates for ROP as Wang et al. in 2019 mentioned that there was a 53% reduction in mortality, especially in the first year of life, from 1990 to 2015 [9]. This is a very reassuring statistic that indicates that this condition is being tackled continuously with the expansion of neonatal healthcare. However, this advancement also comes with an important point that as the healthcare options for neonates with ROP improve steadily, there will be the need to educate and sustain the care for these patients as well. This is very important to note in low- and middle-income countries as, although screening methods may be improved and basic care will be more streamlined with protocol, a lack of resources to sustain this program may result in an increase in the number of children with ROP-related blindness [9,18]. Wang et al. particularly noted that the increased mortality rate seen in Nigeria may have been due to the lack of adequate facilities [9].

A study conducted by Braimah et al. in 2020 [18] in Ghana assessed the aforementioned concept of the income status of the country and its relationship with the incidence, prevalence, and mortality of ROP. This study showed that the overall mortality in this group of patients, born before 37 weeks of gestation and/or having a birth weight less than 2 kg, was 24.6%, and one of the pertinent factors influencing the development of ROP was having a birth weight under 1.5 kg. It was found that this attribute led to an increased likelihood, approximately 30 times more likely, of developing ROP. The study also revealed that an upgrade of facilities in the neonatal intensive care unit (NICU) of a facility in Ghana improved the survival of neonates with a birth weight of less than 2.5 kg from 67.4% to 78.2%.

As mentioned earlier, one of the major risk factors identified for the development of ROP is an early gestational age, specifically neonates born before the gestational age of 32 weeks. It was reported by Edy Siswanto and Sauer in 2017 [16] that the incidence of ROP in neonates of this category increased when the neonatal mortality rate of a country was more than 5. Another risk factor that has been commonly identified in a review of literature includes the unregulated use of oxygen. Results from the SUPPORT Trial and the BOOST-II Trial [12] suggested that there was reduced survival with infants exposed to lower oxygen saturation (SpO2) with a mortality of 17% compared to those exposed to a higher target SpO2 group that had a mortality of 14%. A lower SpO2 target also suggested increased mortality and lower rates of ROP [21].

It is imperative to note that with the advancement of neonatal care, better-implemented screening protocols, and newer treatment modalities being developed for managing ROP, mortality rates have decreased since the disease was first identified. However, in countries such as low- and middle-income countries, implementing a screening protocol means the incidence of diseases such as ROP will increase; however, a sustainable method of management that is economic, effective, and easily implementable must be put into action to actively reduce both the incidence and mortality of ROP.

Current management strategies

In recent times, promising developments have been made with regard to treatment modalities and efficacies for ROP. Many large-scale studies such as the CRYO-ROP, Early Treatment for Retinopathy of Prematurity Study (ETROP), and Bevacizumab Eliminates the Angiogenic Threat of Retinopathy of Prematurity (BEATROP) have led to the formation of current guidelines for managing infants with this disease. For prethreshold type 1 disease, they recommend that laser therapy is a suitable initial step; however, as the condition progresses to an aggressive zone 1 type, anti-VEGF inhibitors such as intravitreal bevacizumab have been shown to provide the most benefit [3]. They are also relatively cheap and abundant in supply throughout the world, making them a popular option for such treatment. According to a randomized controlled trial conducted to evaluate this drug on premature patients, 56 of the 61 study eyes experienced a regression of ROP over a six-month period. The study concluded that the outcomes were very positive for a low-dose treatment. However, since bevacizumab enters the bloodstream, there is a possibility that it can affect the development of other organs. Therefore, further research is needed to evaluate the systemic and long-term impacts of these medications in premature infants [27].

Due to the extensive research and understanding of ROP progression, the current standards of care in the United States emphasize a screening program, in which at-risk infants undergo retinal examinations at specific intervals so that timely treatment can commence. These assessments would ideally be scheduled in accordance with the preterm child’s gestational age at birth, as well as the nature of the disease. The objective of this plan is to detect infants who can benefit from treatment and highlight the need for future appropriate interventions. Such a system would employ the use of wide-angle retinal images and binocular indirect ophthalmoscopes in conjunction with clinical data. The presence of skilled and experienced ophthalmologists is vital for this program, as well as the contributions of neonatologists and imaging staff. Despite the implementation of such screening protocols, there will always remain a small portion of ROP infants that still progress to blindness; however, it should not come as a surprise that presenting late to screening is the most common reason for ROP-related blindness [5,28].

## Conclusions

Retinopathy of prematurity is a preventable disorder of retinal blood vessels that has caused blindness in millions of preterm children throughout the globe. Once it advances into later life, ROP can lead to several complications that include severe myopia, strabismus, cataracts, glaucoma, and retinal detachment. Due to the numerous risk factors of ROP being related to the delivery and early development of preterm infants, this disease can provide interesting insight into the quality of perinatal and postnatal services in a given health center. It should then come as no surprise that poorly developed countries continue to lag behind with regard to ROP prevalence. Fortunately, intriguing breakthrough studies have paved the way for current treatment strategies involving the use of laser therapy and low-dose intravitreal bevacizumab. These emerging methods for the management of ROP provide a considerable reason for optimism moving forward. However, the significance of implementing consistent and strict screening protocols worldwide needs to be highlighted to successfully halt the progression of this potentially debilitating disease.
